# COVID-19 Encephalopathy Presenting As New-Onset Seizure: A Case Report

**DOI:** 10.7759/cureus.28204

**Published:** 2022-08-20

**Authors:** Abdullah Baksh, Alia Hadid, Thiagarajan Jaiganesh

**Affiliations:** 1 Emergency Department, Tawam Hospital, Al Ain, ARE

**Keywords:** sars-cov-2, covid-19, encephalopathy, leptomeningeal enhancement, generalized tonic clonic seizures, altered mental state, emergency medicine physician, icu patients, acute encephalitis

## Abstract

Since its outbreak, it's been well-documented that coronavirus disease 2019 (COVID-19) can present with wide variety of neurological manifestations in absence of the usual respiratory symptoms. We report one such severe neurological manifestation of SARS-CoV-2 infection. To our knowledge, this is the first reported case of COVID-19 encephalopathy with CSF and MRI findings in the United Arab Emirates. We present a case of a 52-year-old female who presented with complaints of altered mentation, anosmia, headache, dizziness, weakness, lethargy, and vomiting. While in the emergency department she developed two generalized tonic-clonic seizure episodes, a more pronounced delirium, and tachypnea which required intubation. She was then admitted to the intensive care unit (ICU). She was COVID-19 positive and subsequent MRI revealed encephalopathy. She was discharged from ICU and was under long-term care at the time of case documentation.

## Introduction

Novel coronavirus infection in the elderly, especially those with other comorbidities, is more likely to render grave illness [1,2]. It is in this population where the greater risk of developing acute encephalopathy and altered mental status lies. We hereby report a case of coronavirus disease 2019 (COVID-19) encephalopathy in which generalized tonic-clonic seizures were the presenting feature of the disease.

## Case presentation

A 52-year-old diabetic female arrived at our emergency department (ED) with a three-day history of headaches, anosmia, dizziness, weakness, lethargy, vomiting, and appetite loss. The patient denied fever, photophobia, neck pain, flu-like symptoms, and shortness of breath. Past medical, surgical, and allergic history was of no significance. She had no history of sick contacts and was not a smoker. On arrival to the ED, she was tachypneic (respiratory rate {RR}: 20 breaths per minute {bpm}), tachycardic (heart rate {HR}: 117 beats per minute {bpm}), was saturating well on room air (SpO_2_: 99%), and had a Glasgow Coma Scale (GCS) of 14/15. General cardiorespiratory and neurological examination was otherwise unremarkable.

**Figure 1:**
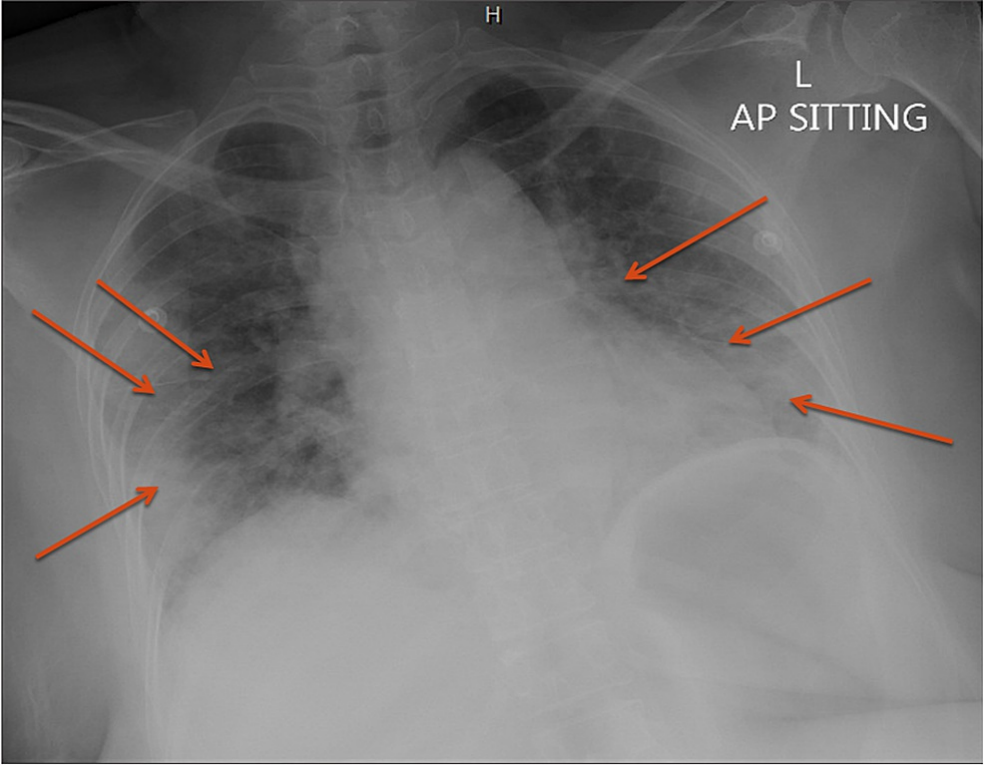
Chest x-ray showing bilateral pulmonary consolidations. AP: anteroposterior

The initial impression was pneumonia with respiratory distress based on elevated inflammatory markers and chest x-ray finding of bilateral pulmonary consolidations (Figure 1). The patient was empirically treated with antibiotics as per local protocols.

**Figure 2:**
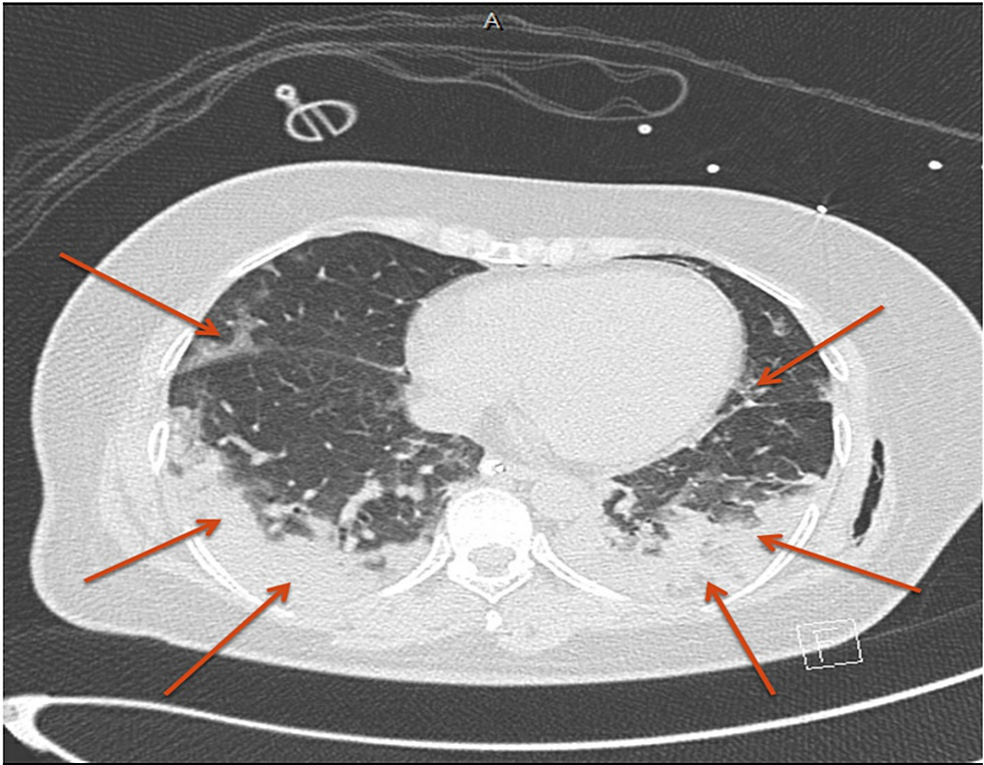
CT thorax showing bilateral basal large consolidations associated with multifocal peripheral ground glass opacities, typical for COVID-19 pneumonia.

Twenty minutes later, the patient developed two episodes of generalized tonic-clonic seizures and required intubation. CT thorax showed features typical of COVID-19 pneumonia with a severity score of moderate (Figure 2).

COVID-19 nasopharyngeal swab test (performed by the MDx 2019 n-COV reverse transcription polymerase chain reaction {RT-PCR}) was positive and COVID-19 prognostic markers were all elevated (Table 1). 

**Table 1:** COVID-19 prognostic markers as per hospital protocol. LDH: lactate dehydrogenase; CRP: c-reactive protein; COVID-19: coronavirus disease 2019; HI: high

Prognostic markers	Reference range and units	Initial results
LDH	135-214 IU/L	476 IU/L (HI)
Ferritin	30-400 mcg/L	1,185 mcg/L (HI)
D-dimer	0.129-0.523 mg/L	28.75 mg/L (HI)
Troponin T	<14 ng/L	104.5 ng/L (HI)
CRP	<5.0 mg/L	151.8 mg/L (HI)
Interleukin-6	≤7.0 pg/mL	124 pg/mL (HI)

The patient was admitted to the ICU as a case of COVID-19 pneumonia with sepsis and possible secondary meningitis, encephalitis, and/or encephalopathy. Following her positive COVID-19 nasopharyngeal swab result, she was started on COVID-19 therapy as per local protocol which, at the time, included hydroxychloroquine sulfate and favipiravir.

A full workup, including CT head, was done to rule out other correctable causes of seizures, and they were all unremarkable. Given the picture of high inflammatory markers, new seizure activity, and a normal CT brain, lumbar puncture was done and she was empirically treated with acyclovir, ceftriaxone, and vancomycin for meningoencephalitis.

Subsequently, lumbar puncture result revealed a WBC count of 10/mm^3^ with a lymphocyte predominance (73%), high protein (0.57 g/L), and glucose (124 mmol/L), and a negative CSF culture, pointing to encephalopathy rather than encephalitis (Table 2).

**Table 2:** Cerebrospinal fluid (CSF) analysis, as per hospital protocol.

CSF analysis	Reference range and units	Initial results
Opening pressure	60-250 mmH_2_O	71 mmH_2_O
Color	Colorless	Colorless
Appearance	Clear	Clear
WBC	0-5/mm^3^	10/mm^3^ (HI)
RBC	<1/mm^3^	106/mm^3 ^(HI)
Glucose	2.2-3.88 mmol/L	124 mmol/L (HI)
CSF: S_Glu_	>0.6	1.01
Protein	0.15-0.45 g/L	0.57 g/L (HI)
Cytology	50-70% lymphocytes	Dominated by lymphocytes (73%) with few monocytes and neutrophils seen
Culture	No growth	No growth at all
Gram stain	No organisms seen	No organisms seen

**Figure 3:**
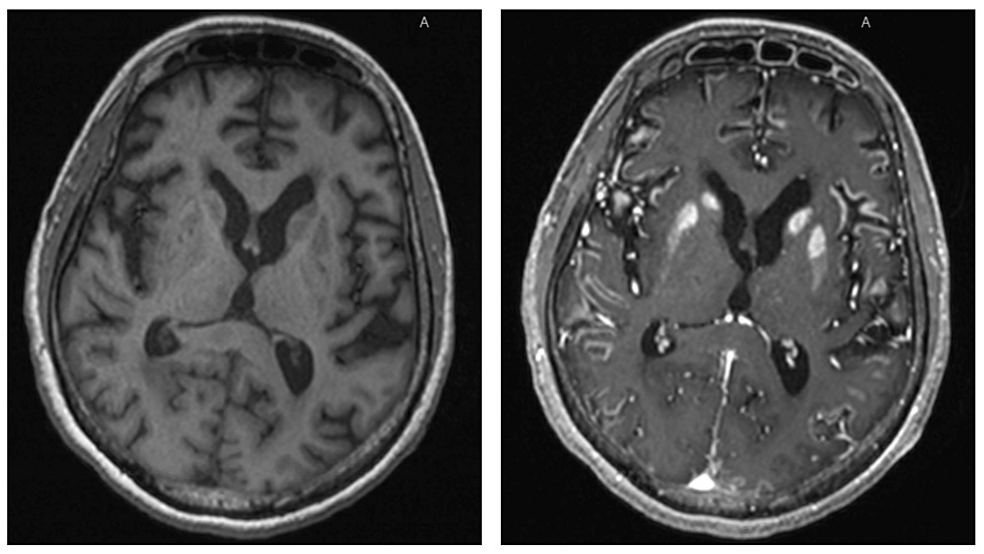
MRI brain scan showing extensive leptomeningeal enhancement and bilateral symmetrical basal ganglia enhancement. T1-weighted image pre-contrast (left) and T1-weighted image post-contrast (right).

Due to unavailability of CSF PCR analysis, presence of COVID-19 virus in CSF could not be ruled out. During the course of the ICU stay, GCS remained 3/15 without sedation, so status epilepticus was suspected and treatment with levetiracetam and lacosamide was started. EEG could not be done because of risk of exposure as per hospital protocols. The repeat CT brain was unremarkable, and she was scheduled for an MRI of the brain. MRI brain revealed extensive leptomeningeal enhancement and bilateral symmetrical basal ganglia enhancement, suggestive of meningoencephalopathy (Figure 3).

Eventually, given that her Glasgow Coma Score (GCS) remained 4/15 off paralytics and sedatives, tracheostomy was done and she was shifted to a long-term care facility once she became COVID-negative, where she resides to date.

## Discussion

Coronavirus disease 2019 (COVID-19) caused by SARS-CoV-2 has neurotropic and neuroinvasive abilities. Neurological problems in patients with coronavirus infection range from disorders of smell and taste to encephalopathy, seizures, and strokes, as reported by numerous studies [3]. Our report shows yet another case of COVID-19-associated neurological manifestations, mainly encephalopathy.

The mechanism by which SARS-CoV-2 causes encephalopathy is currently unknown. The most commonly accepted notion is the hematogenous spread of viral particles into the CNS via circulating lymphocytes [4]. Another theory is viral movement in a retrograde fashion across the cribriform plate into the CNS [4]. In addition to viral infection, host immune response causing a cytokine storm leading to damage in the blood-brain barrier, increasing leukocyte migration and inflammation, maybe another mechanism causing encephalopathy [4]. It has been proposed that SARS-CoV-2 can infiltrate the brainstem leading to dysfunction of the cardiorespiratory centers of the brainstem, which explains the deterioration of respiratory effort and a subsequent need for ventilation [5].

Amid the numerous common presentations of COVID-19, a seizure is an uncommon initial neurological presentation [6]. The most extensive retrospective case series encompassing 1,043 COVID-19-positive admitted patients found that only four (0.4%) of the patients presented with new-onset seizures [7].

Leptomeningeal enhancement on MRI is an essential differentiator between a diagnosis of COVID-19 encephalopathy and hypoxic encephalopathy. As in our case, leptomeningeal enhancement was noted in eight out of 13 patients undergoing MRI due to unexplained encephalopathy features [8].

Another case series provided evidence of hypoxic brain damage detected on MRI, which revealed T2-fluid attenuated inversion recovery (FLAIR) hyperintensities, and came to the conclusion that hypoxic damage more frequently presents with these findings on MRI rather than leptomeningeal enhancement induced directly by SARS-CoV-2 [9].

Due to the unavailability (CSF PCR analysis) and concerns regarding the spread of infection, electroencephalogram (EEG) was not done. However, SARS-CoV-2 CSF PCR analysis has been found to be specific [10], but not sensitive in detecting direct viral invasion of the central nervous system [11,12]. Also, there is a lack of evidence supporting specific stereotyped EEG patterns in patients with COVID-19 encephalopathy, nor are any abnormal waveforms predictive of a neurological prognosis for this population [13,14].

As compared to general COVID-19 therapies including but not limited to glucocorticoids, remdesivir, interleukin-6 (IL-6) pathway inhibitors, and other antibody therapies [15], the management of COVID-19 encephalopathy, per se, remains to be entirely supportive, with treatment being directed towards the underlying cause.

Our patient presented early in the pandemic when hydroxychloroquine and favipiravir were considered a potential treatment modality. She did not receive glucocorticoids or immunomodulator therapy which were later studied and recommended [15]. There are reported case series where patients with severe encephalopathy improved clinically after receiving corticosteroids, immunotherapy, and plasma exchange [16,17]. There are similar reported cases of COVID-19 encephalopathy, where pulse therapy of high-dose corticosteroids showed promising results [16,18]. However, further research is needed to establish the effectiveness and responsiveness of corticosteroid therapy in encephalopathy [16].

COVID-19 encephalopathy patients tend to have increased morbidity and mortality, similar to our case [19]. One study reported that COVID-19 encephalopathy patients had longer durations of hospital stay, worse functional impairment at discharge, and higher 30-day mortality rate compared to COVID-19 patients without encephalopathy (22% vs 3%) [19]. Further long-term outcomes remain to be elucidated.

## Conclusions

The diagnosis of COVID-19 encephalopathy was established for our patient based on the fact that her neurological status declined despite the improvement of her general condition, and other major causes of CNS affection were ruled out. COVID-19 encephalopathy is potentially a severe neurological manifestation of the SARS-CoV-2 infection and due to its highly variable presentation, clinicians need to have a high index of suspicion. To better understand the neuropathological capabilities of COVID-19, its long-term impact on patients with pre-existing comorbidities and further treatment modalities, ongoing investigations are recommended.
